# Doughnut Shaped Parathyroid Adenoma

**DOI:** 10.4274/mirt.galenos.2018.06977

**Published:** 2019-03-19

**Authors:** Derya Çayır, Mehmet Bozkurt, Mehmet Erdoğan, Salih Sinan Gültekin, Cem Azılı, Ata Türker

**Affiliations:** 1 University of Health Sciences, Dışkapı Yıldırım Beyazıt Training and Research Hospital, Clinic of Nuclear Medicine, Ankara, Turkey; 2 Süleyman Demirel University Faculty of Medicine, Department of Nuclear Medicine, Isparta, Turkey; 3 University of Health Sciences, Dışkapı Yıldırım Beyazıt Training and Research Hospital, Department of General Surgery, Ankara, Turkey; 4 University of Health Sciences, Dışkapı Yıldırım Beyazıt Training and Research Hospital, Department of Pathology, Ankara, Turkey

**Keywords:** Parathyroid adenoma, Tc-99m sestamibi, SPECT

## Abstract

A 52-year-old woman presented with a complaint of neck swelling. The patient showed signs of hyperparathyroidism: hypercalcemia, and hypophosphatemia. Tc-99m MIBI dual-phase parathyroid scintigraphy and SPECT revealed increased activity in a regular-bordered, “doughnut”-shaped mass on the left side of the thyroid gland with a central hypoactive area. The cervical ultrasound identified a mixed echoic thyroid nodule with a central large cystic portion, and no parathyroid gland abnormality. Total thyroidectomy and parathyroid exploration was performed. Pathological evaluation of the resected thyroid specimen reported a giant intra-thyroidal hemorrhagic parathyroid adenoma.

## Abstract (tr)

Boyunda şişlik şikayeti ile başvuran elli iki yaşında kadın hastada hiperkalsemi, hipofosfatemi ile hiperparatiroidizm saptandı. Yapılan Tc-99m MIBI dual faz paratiroid sintigrafisinde ve SPECT çalışmasında tiroid bezinin sol lobunu kaplayan, ortasında hipoaktif alanlar izlenen, artmış aktivite tutulumu gösteren “doughnut” görünümlü lezyon izlendi. Boyun ultrasonografide sol lobun üst ve orta kesiminde ortasında kistik komponent görülen, karışık ekoda tiroid nodülü gözlendi, ancak paratiroid patolojisi izlenmedi. Hastaya total tiroidektomi ve paratiroid eksplorasyonu yapıldı. Tiroid cerrahi spesimeninin patolojik deperlendirmesi dev intratiroidal hemorajik paratiroid adenomu olarak raporlandı.

## Figures and Tables

**Figure 1:**
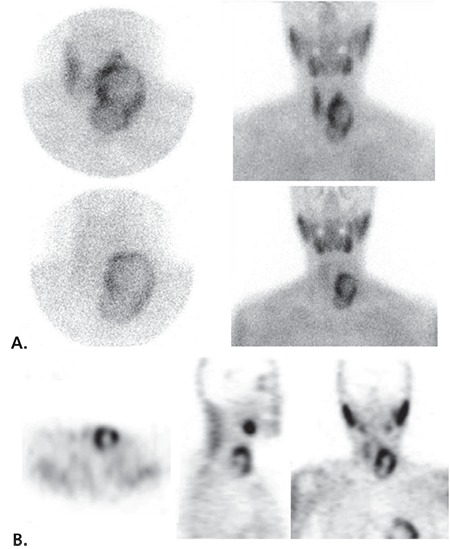
A 52-year-old woman presented with a complaint of neck swelling. The patient’s laboratory examinations showed high levels of serum parathormone [356.5 pg/mL (normal range: 12-88)], hypercalcemia [12.37 mg/dL (normal: 8.8-10.6)], and hypophosphatemia [2.29 mg/dL (normal: 2.5-4.5)]. Primary hyperparathyroidism is the most frequent reason of hyperparathyroidism, and the most common cause of hyperparathyroidism is solitary parathyroid adenoma (1). Tc-99m MIBI parathyroid scintigraphy and cervical ultrasound (US) are the methods of choice for parathyroid imaging (2), while Tc-99m MIBI parathyroid scintigraphy shows good correlation with parathyroid hormone level and histopathologic diagnosis (3). Accordingly, we performed Tc-99m MIBI dual-phase parathyroid scintigraphy (A) and SPECT (B), on which an increased activity including a central hypoactive area as a regular round doughnut-shaped mass on the left side of the thyroid gland, extending through inferior part of the neck, was observed.

**Figure 2:**
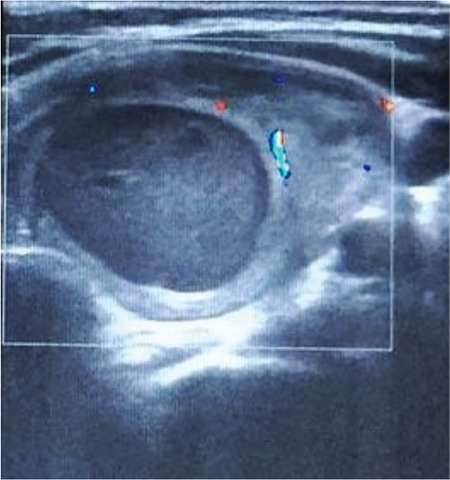
After finding out this MIBI active mass, cervical US was carried out to identify the lesion characteristics. The US revealed a mixed echoic intrathyroidal lesion, with a polar vascularity on color doppler US that was 36 mm in dimension with a central large cystic portion. The curative treatment for primary hyperparathyroidism is the surgical excision of the hyper-functioning parathyroid tissue (4).

**Figure 3:**
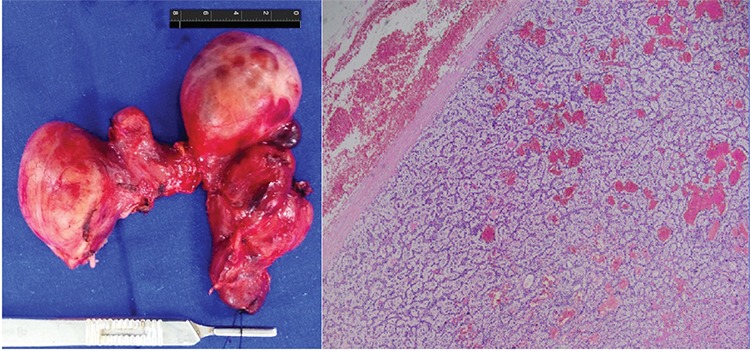
Consequently, the patient underwent total thyroidectomy and parathyroid exploration. Pathologic evaluation of the resected thyroid specimen revealed parathyroid adenoma of about 8 cm in diameter with extensive bleeding, localized within the left lobe. The prevalence of intrathyroidal parathyroid adenoma is around 1% in surgical series (5), and giant intrathyroidal parathyroid adenomas are extremely rare (6). Whenever the diagnosis of a parathyroid adenoma is in question, Tc-99m MIBI dual-phase scan and SPECT or SPECT/CT can help to identify the parathyroid adenoma in patients with hyperparathyroidism.
